# The adipose tissue keeps the score: priming of the adrenal-adipose tissue axis by early life stress predisposes women to obesity and cardiometabolic risk

**DOI:** 10.3389/fendo.2024.1481923

**Published:** 2024-10-18

**Authors:** Meghan Blair Turner, Carolina Dalmasso, Analia S. Loria

**Affiliations:** Department of Pharmacology and Nutritional Sciences, University of Kentucky, Lexington, KY, United States

**Keywords:** adverse childhood experience, sex differences, obesity, aldosterone, HPA axis

## Abstract

Adverse Childhood Experiences (ACEs) refer to early life stress events, including abuse, neglect, and other psychosocial childhood traumas that can have long-lasting effects on a wide range of physiological functions. ACEs provoke sex-specific effects, whereas women have been shown to display a strong positive correlation with obesity and cardiometabolic disease. Notably, rodent models of chronic behavioral stress during postnatal life recapitulate several effects of ACEs in a sex-specific fashion. In this review, we will discuss the potential mechanisms uncovered by models of early life stress that may explain the greater susceptibility of females to obesity and metabolic risk compared with their male counterparts. We highlight the early life stress-induced neuroendocrine shaping of the adrenal-adipose tissue axis as a primary event conferring sex-dependent heightened sensitivity to obesity.

## 1 Introduction

Adverse childhood experiences (ACEs) are traumatic events that happen before the age of 17, such as abuse, neglect, parental separation, and household instability. Cumulative number of ACEs have been associated with an increased risk for premature death (1, 2). ACEs are highly prevalent worldwide, as six out of 10 adults report experiencing at least one adverse event in childhood (3). Based on 2011–2020 Behavioral Risk Factor Surveillance System (BRFSS) data, the CDC estimates that the prevalence of ACEs among U.S. adults is similar. Overall, 63.9% of U.S. adults reported at least one ACE and 17.3% reported exposure to four or more ACEs (4). In 1998, a large health maintenance organization clinic in San Diego published a seminal study on ACEs. This report revealed that increased ACE exposures corresponded to increased rates of severe obesity, ischemic heart disease, and liver disease (1). Specifically, participants who experienced more than three ACEs had a 1.5 to 1.9-fold risk for severe obesity (Body Mass Index, BMI, ≥ 35 kg/m^2^) in adulthood (1, 5).

Since that publication, numerous epidemiological and population-based studies have replicated the link between ACE exposures and increased BMI, obesogenic behaviors, and cardiometabolic risk factors in adulthood. A recent systematic review found that four or more ACE exposures results in increased risk for obesity, diabetes, heart disease, and risk-taking behaviors (6). Moreover, a study of Finnish adolescents found that multiple ACE exposures confer a 46% increased likelihood of adult obesity (7), while The Growing Up in Ireland cohort found that ACE exposure before age nine was associated with early adolescent obesity (8). Also, ACE exposure was found to be associated with type 2 diabetes, whereas neglect has the strongest influence increasing the risk for metabolic disease (9). The seminal ACE study was based on a retrospective survey comprised of 10 binary questions regarding childhood physical and emotional abuse, however, validated variations are widely used in clinical and epidemiological settings (1, 10). Notably, a cumulative effect of ACE exposure was demonstrated, as each positive response on the ACE questionnaire corresponded to a one-point increase in BMI (10).

Other factors such as sex, race, and type of ACE exposure also contribute to the stratification of the effects of ACEs on BMI and metabolic dysregulation. The Trondelag Health Study in Norway found that parental divorce and negative childhood memories were associated with higher women’s pre-pregnancy BMI (11). In a longitudinal U.S. cohort of women, two or more ACE exposures corresponded to increased adolescent and adult BMI, where sexual and physical abuse specifically affected Black women (12). The UK-based Millennium cohort found that BMI in females was increased by a single ACE exposure, and was most sensitive to parental separation and physical punishment, while in males, elevated BMI emerged after the occurrence of three or more ACEs (13). The number of ACEs needed to increase BMI in both Black and white women was reduced compared with white men (14). In contrast, Black men showed a negative association between ACEs and BMI; however, increased waist circumference suggested greater central adiposity (14).

Animal models have recapitulated the sex-specific effects of early life stress on metabolic function and body composition, providing a translational tool to identify therapeutic targets and effective approaches to treat disease in this vulnerable population. Modeling ACEs in rodents is possible based on the premise that the mammalian maternal bond is crucial for modulating the stress response in early life (15). Critical windows of stress susceptibility have been observed across mammalian species, from rodents to non-human primates, as well as in human infants, which are protected by maternal sensory input (16). In rodents, a stress hypo-responsive period (SHRP) that occurs during the first two postnatal weeks is characterized by lower corticosterone levels and corticosterone unresponsiveness (16, 17). This SHRP is critical for the optimal development and maturation of the brain and other physiological systems. Thus, high levels of stress hormones during this critical period result in the long-term dysregulation of the stress response (18–21).

Investigations of the effects of early life stress have found significant impairment of the adrenal-adipose tissue axis function in women and pre-clinical models using female rodents. Notably, aldosterone has emerged as an adrenal hormone associated with a sex-specific cardiometabolic risk, where females with early life stress experience increased susceptibility (22–24). In women, plasma aldosterone correlates positively with visceral adipose tissue and negatively with insulin sensitivity, and these associations are independent of plasma renin activity (25, 26). It is known that aldosterone synthesis is stimulated by adrenocorticotrophic hormone (ACTH), arginine-vasopressin (AVP), angiotensin II (Ang II), leptin, and potassium (27). Subsequently, high circulating aldosterone binds to mineralocorticoid receptors expressed in adipocytes, immune cells, endothelial cells, and brain areas controlling the metabolic function. Along these lines, female mice exposed to early life stress have shown increased plasma and adrenal-derived aldosterone, as well as a mineralocorticoid-dependent expansion of adipose tissue and metabolic dysregulation (24, 28, 29). Early life stress exposure does not increase basal adrenal glucocorticoids in adult women or rodents (24, 30); however, corticosterone response to stress is exacerbated (31). Adrenal androgens are not well-studied in this context due to limited translational potential, as rodents do not display a defined period of adrenarche and secrete adrenal androgens in minuscule quantities (32). Additionally, adrenal androgens are difficult to study in humans, as gonadal androgens are systemically prominent (32).

In his New York Times best-selling book, *The Body Keeps the Score*, Dr. Bessel van der Kolk discusses how traumatic experiences commonly induce neurobiological, immune, and cardiovascular changes in the body. However, we put forth that in women, the adipose tissue is truly the scorecard marked upon by early life stress, as the dysregulated hypothalamic-pituitary-adrenal (HPA) axis predisposes to obesity and metabolic syndromes.

## 2 Early life stress models

Animal models that recapitulate the long-term effects of early life stress aim to disrupt or limit access to maternal care during early postnatal life. In rodents, this is commonly achieved by separating the mother from her litter of pups during the SHRP, which is known as maternal separation (MS) (33, 34). A more aggressive model of neglect in early life is to combine MS and early weaning (MSEW) (28, 35). This model is widely used in mice because murine offspring exhibit greater stress resilience than rats. Maternal care disruptions induce a stress response during a time when the HPA axis is not fully developed, which programs the sensitivity threshold for subsequent stressors. As such, maladapted HPA axis sensitivity is a hallmark of early life stress models that leads to neuroendocrine, behavioral, and cardiometabolic outcomes that mimic those observed in children and adults exposed to ACEs (36).

Different MS paradigms exist that involve removing the dam from the whole litter in the home cage, for durations ranging from 10 minutes to 3- 8 hours daily (34). Across MS models, separations are typically performed daily for 3-14 days, either consecutively or intermittently. Litters from an undisturbed cage often serve as a control group, while in other studies, half of the litter identified by tail snip or ink marks is subjected to separations and the other half remains with the dam, serving as controls. MS is typically sufficient to induce long-term HPA disruptions in rats; however, MSEW is a more effective approach to induce long-lasting neuroendocrine dysfunctions in mice (35). In MSEW paradigms, pups are not only separated from their mothers, typically for longer periods but are also weaned prematurely at postnatal day (PND)17 compared to controls weaned at PND21 (28, 35). Another paradigm of early life stress involves maternal deprivation (MD), a form of MS where offspring are separated individually for up to 24 hours, which induces higher levels of stress on the pups (20, 37). When pups are subjected to MD for shorter periods, the protocol can also be referred to as early handling (15 minutes) or early deprivation (four hours) (38).

Maternal care disruption induced by MS or MSEW promotes fragmented nursing, anxiety, depression, aggression, and changing licking/grooming frequency that have been shown to sensitize the offspring’s response to subsequent stressors in the short and long term (39–42). Another model of early life stress uses this approach by providing limited nesting and bedding (LNB) materials for the dam. The LBN model leads to disorganized maternal care in addition to poor nest construction which disrupts thermoregulation, an environmental stressor affecting the dam and pups (41, 43).

## 3 Early life stress and the HPA axis

**Figure 1:**
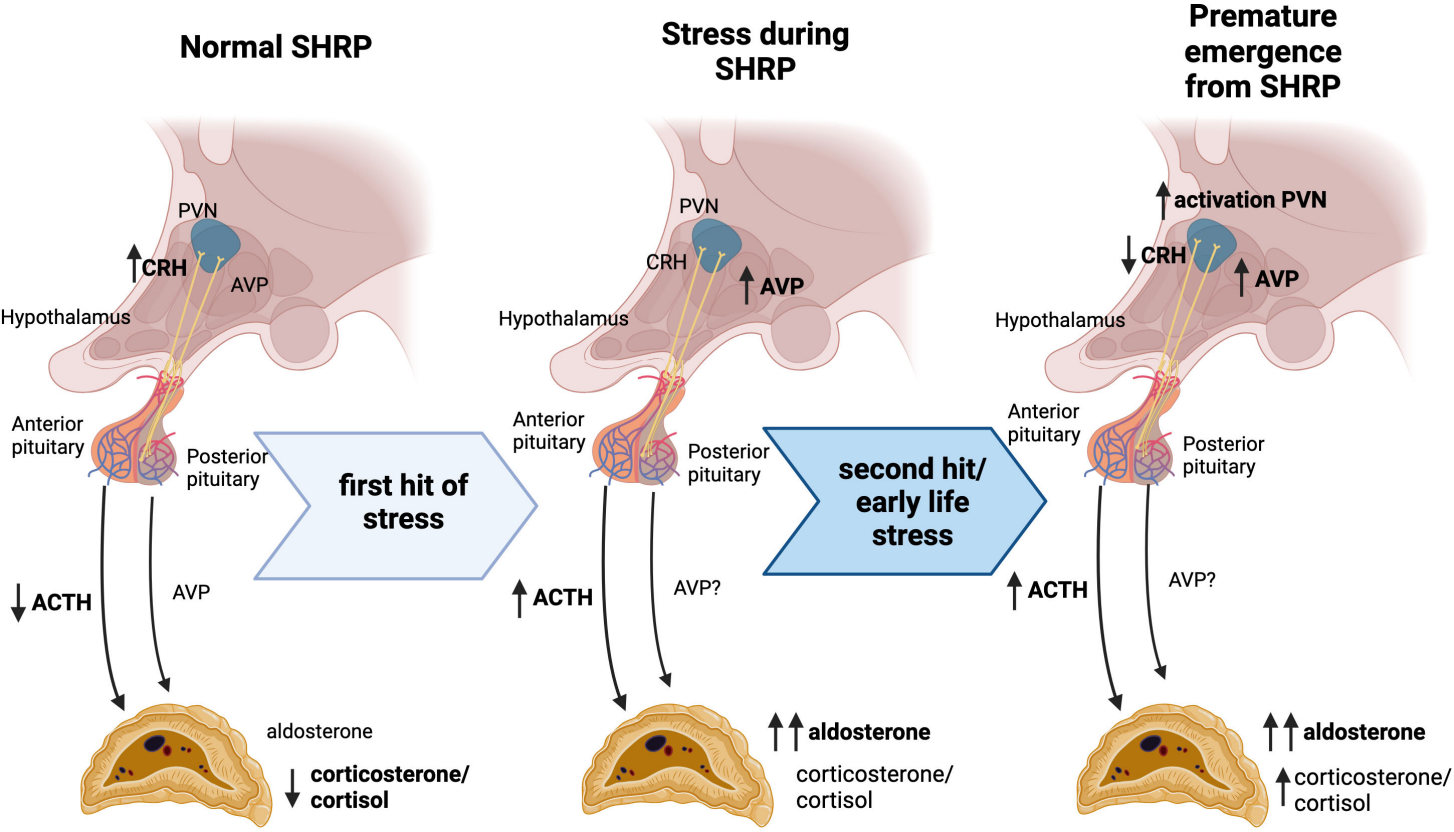
Progressive stress exposure during the stress hyporesponsive period alters HPA function. In rodent models, SHRP is characterized by decreased ACTH and corticosterone production despite increased CRH expression in the hypothalamus. A first hit of stress during this period elicits a modest elevation in AVP and ACTH, coupled with a significant increase in aldosterone. After early life stress exposure, a subsequent hit of stress leads to increased activation in the PVN accompanied by increased AVP expression and decreased CRH expression. This “second hit” elicits an increase in ACTH, followed by elevated corticosterone and significant increases in aldosterone. Priming of the HPA axis during this period of altered stress response mechanisms may underlie the predisposition for a hyper-aldosteronogeneic response to stressors such as high fat diet in adulthood.

Activation of the HPA axis is triggered by stressful events that induce the release of corticotropin-releasing hormone (CRH) and AVP from magnocellular neurons in the paraventricular nucleus of the hypothalamus (PVN) to the pituitary gland (**Figure 1**). CRH stimulates ACTH synthesis and release into the bloodstream in the anterior pituitary gland, while AVP is released directly into the portal veins of the posterior pituitary (**Figure 1**). Upon stimulation, both hormones are released into the systemic circulation and bind to specific receptors in the adrenal glands to promote rapid corticosteroid synthesis (27). ACTH binding to melanocortin type 2 receptors (Mc2R) results in calcium-mediated increase in transcription and activity of rate-limiting steroidogenic acute regulatory protein (StAR) (44, 45). AVP binds to AVP receptor type 1 (V1R), inducing a similar intracellular signaling pathway (46, 47). StAR is responsible for supplying the steroidogenic substrate, cholesterol, to the mitochondrial membrane for downstream conversion to steroid hormones based on cell-specific enzymes (44). In cells of the *zonas fasciculata* and *glomerulosa* of the adrenal gland, enzymes, including CYP11B1, are present for the synthesis of corticosteroids, corticosterone, and cortisol, specifically in humans (48). However, activation of CYP11B2 transcription in the *zona glomerulosa*, further converts corticosterone into the primary mineralocorticoid, aldosterone (49). This pattern of enzyme expression defines the zone-specificity of glucocorticoid and mineralocorticoid production (50).

The SHRP is marked by a relatively quiescent stress response compared to immediately after birth or following the SHRP (16, 17), and is well documented to occur between PND4-14 in rats and PND1-12 in mice (16, 21, 51, 52). Expression of CRH in the PVN is exceptionally high during this time, only downstream ACTH and corticosterone release in response to mild stress are dampened (20, 21) (**Figure 1**). Sex differences in HPA function are in part due to differences in the circulating gonadal steroid hormone. While testosterone seems to inhibit HPA function, estrogens show stimulatory effects. One mechanism by which androgens and estrogens modulate stress responses is through the binding to their cognate receptors in the central nervous system showing sex and site-specific distribution. In the case of androgens, data suggest that the control of the hypothalamic paraventricular nucleus is mediated trans-synaptically. For estrogen, modulation of the HPA axis may be due to changes in glucocorticoid receptor-mediated negative feedback mechanisms (53). Glucocorticoid-receptor-mediated negative feedback suppresses ACTH response to mild stressors. However, if the stressor is sufficient to reach a threshold the neonatal pituitary is able to producing a robust ACTH response (16).

Exposure to a more severe stressors during early postnatal life can cause premature emergence from the SHRP (**Figure 1**). For instance, although early life stress induces an attenuated CRH expression in the PVN, plasma corticosterone remains elevated (19, 20, 54). Artificially-reared PND12 rat pups remained hypo-responsive as long as rat milk substitute was supplemented via gastrostomy (55). Dietary glucose supplementation or blocking ghrelin signaling were also able to attenuate MS effects (56). Interestingly, early life stress did not alter body weight or growth at weaning (24, 28, 55). Taken together, this data indicates that hunger-induced stress could play a critical role in the metabolic programing of the obesogenic response regardless significant effects in growth and weight gain.

Most studies of early life stress focus on corticosterone as the major output of the HPA-mediated stress-response; however, pituitary hormones are also potent stimulators of aldosterone. Rat pups subjected to lipopolysaccharide and hypoglycemic challenges during the SHRP show increased circulating aldosterone compared to non-challenged pups or adults. This increase in aldosterone levels cannot be attributed to elevations in ACTH alone (57). Additionally, primary adrenal cultures from MD rat pups produce more aldosterone than cultures from non-separated pups; however, corticosterone production is not different between groups (58). Early life stress has been shown to methylate the AVP enhancer, leading to increased expression in adult rodents (59, 60), and AVP is clinically elevated in adults with early life stress exposures (61). Taken together, these data suggest that AVP may play a role in mediating increased aldosterone production following early life stress exposure.

Activation of the PVN is similar between maternally deprived and non-deprived pups after a “second hit” of restraint stress; however, maternally deprived pups show reduced CRH and increased AVP expression in this brain area (20). This suggests that deprivation shifts the hypothalamic stress-response from primarily CRH-mediated to AVP-mediated. Neither AVP nor CRH, however, entirely account for steroidogenic stimulation in early postnatal rats (62, 63). Taken together, these studies suggest that while the anterior pituitary and *zona fasciculata* stress responses are dampened during the SHRP, the posterior pituitary and *zona glomerulosa* are preferentially activated during this period. These insights open up the discussion concerning whether the early neonatal period may not be hypo-responsive at all, rather the stress response has been historically measured in the context of a mature neuroendocrine system. Further, priming of these systems in early life leads to long-term differential activation that can be pathogenic during adult life.

## 4 Sex differences in the HPA axis

**Figure 2:**
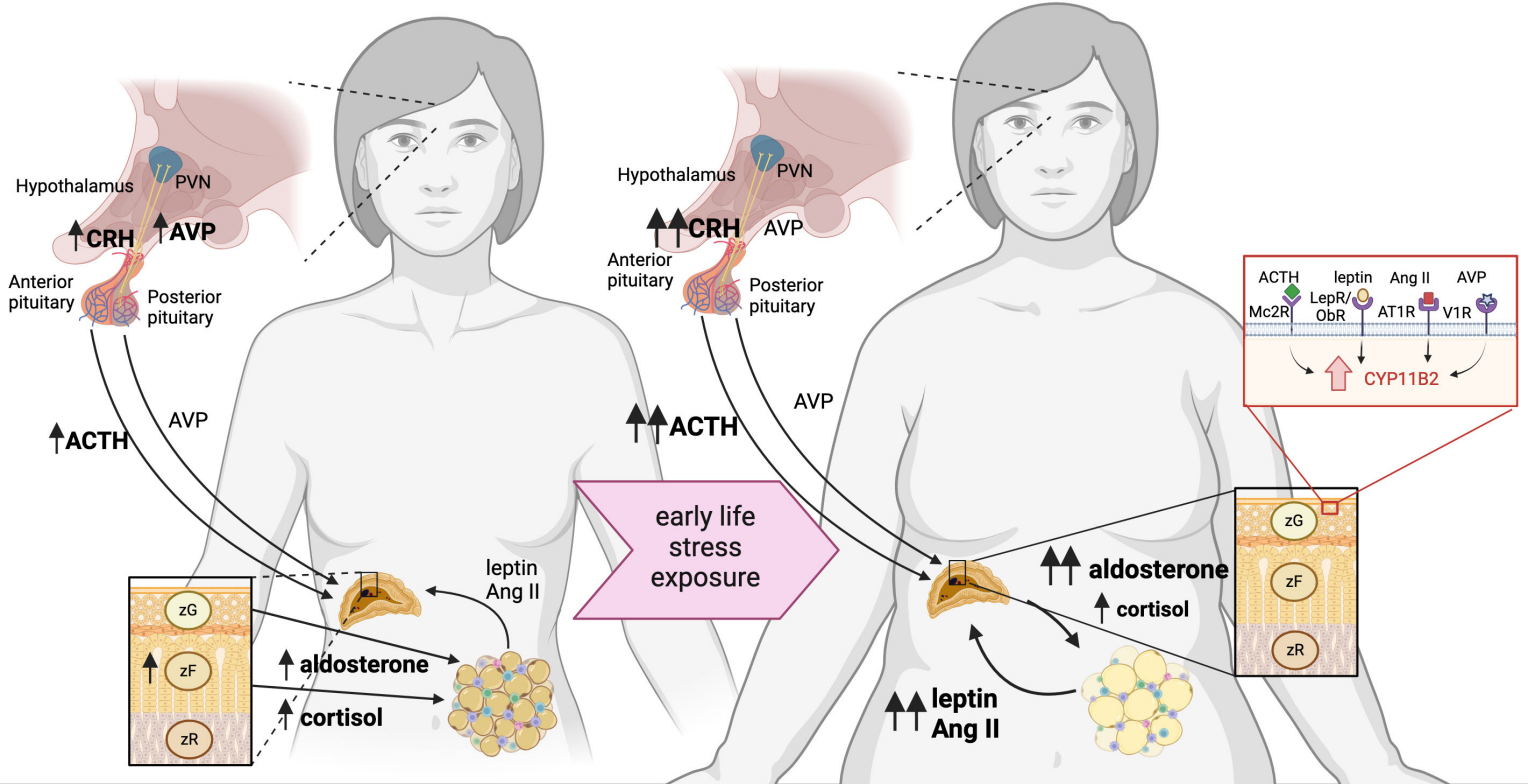
Neuroendocrine mechanism linking early life stress with increased susceptibility of women to obesity. Women have heightened HPA axis activation and responsiveness, which is exacerbated by early life stress, predisposing to obesity. Females have increased activation of the hypothalamus, delineated by increased CRH and AVP expression, compared to males. Both women and female rodents have elevated ACTH in plasma, as well as aldosterone and cortisol/corticosterone. Increased cortisol/corticosterone is attributed to an expanded *zona fasciculata* in the adrenal glands, which are larger in females than males. Exposure to early life stress leads to sensitization of the HPA axis, which increases CRH in the hypothalamus of rodent models and increased plasma ACTH and AVP in women and female rodents. These secretagogues elicit an elevated basal corticosterone/cortisol, but moreso, elevated aldosterone from the adrenal glands via binding to MC2R and V1R. Adrenal aldosterone acts on the mineralocorticoid receptor in adipose tissue to exacerbate production of adipokines leptin and AngII production, which stimulate further aldosterone production via LepR and AT1R, respectively.

Sexually dimorphic responses are documented in a wide variety of stressors. Female rodents generally display increased baseline HPA axis activity, and exaggerated HPA responses to stress (64). In a resting state, females have increased CRH mRNA expression in the PVN (65, 66) and higher circulating corticosterone compared to male counterparts (66–68) (**Figure 2**). Although stress increases the CRH expression in the PVN in both males and females, the magnitude and duration of the response are greater in females (69, 70). Female mice are also more sensitized to CRH-induced ACTH production (71, 72), and show elevated AVP expression in the stress-responsive medial parvocellular dorsal division of the PVN (66, 70) (**Figure 2**). Despite that females display increases in CRH and AVP synthesis in response to stressful stimuli (73), differences in neuronal activation are rarely reported (66, 74, 75).

Both the total size and size relative to body mass of the adrenal gland are increased in females, which is attributed to hyperplasia of the corticosterone-producing *zona fasciculata (* 76, 77). Furthermore, the female adrenal also produces more corticosterone in response to *in vivo* ACTH stimulation compared to adrenal-derived production in males (67, 78). Both central and peripheral mechanisms have been shown to sensitize the stress response in female rodents and humans. Additionally, female rodents have an increased capacity to synthesize adrenal steroid hormones peripherally, even at resting conditions.

Gonadectomy studies have elucidated striking and consistent effects of sex hormones on the developmental organization and function of the HPA axis. Testosterone is overall protective against HPA axis activation, whereas orchidectomized rodents display increased basal CRH expression in the PVN (79–81) and plasma corticosterone (70, 82, 83) compared to intact males. Stress-induced CRH expression (70) and plasma ACTH and corticosterone levels (83–87) are also elevated after testis removal. Conversely, testosterone administration reverses the effects of gonadectomy, dampening HPA activation (83, 85–87). Interestingly, orchidectomy does not appear to alter the sensitivity of the pituitary to CRH or the adrenal to ACTH (85, 87). On the other hand, female sex hormones, primarily estrogen and progesterone, tend to exacerbate the activation of the HPA axis. Ovariectomy leads to decreased adrenal weight (88, 89) and reduced HPA output (71, 87, 90, 91) while estrogen or estrogen combined with progesterone increases ACTH-stimulated corticosterone responses in male and ovariectomized female rodents (92–94). Similarly, estradiol treatment in men leads to increased ACTH and cortisol in response to psychological stress (95). Gonadal hormones have broad and well-studied effects on all aspects of the HPA axis, which extend beyond the scope of this review but are extensively covered elsewhere (64).

## 5 Early life stress mediates sex-specific cardiometabolic outcomes in adulthood

The average American diet is high in both calories and fats, which is associated with the ever-rising obesity epidemic (96). In rodents and humans exposed to early life stress, hypercaloric diet acts as a secondary metabolic stressor. Dalmasso et al. discovered that male rodents exposed to MSEW and fed a HFD, display fat-derived sympathoextitatory contributing to the exacerbated neurogenic hypertension, despite showing similar levels of adiposity compared to male controls (97, 98). The mechanism underlying the increases in blood pressure in this model involves the activation of the PVN in response to the stimulation of gonadal fat-derived afferent signals, a mechanism referred as adipose-afferent reflex. Unlike males, female MSEW mice fed a HFD develop an exacerbated cardiometabolic phenotype that is independent of the overactivation of the sympathetic system (24). Female MSEW mice weaned to a HFD display increased fat mass in adulthood compared to normally reared and diet-matched controls (29). Further, this sex-specific increases in adiposity were due to greater adipocyte size and triglyceride deposition compared to controls (24). Obese MSEW female mice also showed reduced glucose tolerance, and increased expression of adipogenic genes in pre-adipocytes including the glucocorticoid receptor (Nr3c1), peroxisome proliferator-activated receptor alpha (Pparα), and fatty acid binding protein 3 (Fabp3), and dramatic downregulation of aquaporin 3 (Aqp3), a glycerol efflux channel, and increased hepatic lipogenic gene expression (24, 28, 29, 99).

As reported in female mice, female rats subjected to MS showed increased adiposity in response to a chronic HFD (99). This phenotype was reverted by metyrapone administration prior to the daily MS exposure, inhibiting both corticosterone and aldosterone synthase enzymes. This data supports the notion that blunting adrenal corticosteroid production in response to early life stress prevents the long-lasting effects on the metabolic adaptations to HFD. Additional studies are needed to elucidate the role of the adrenal-adipose tissue axis as a mechanism by which more severe cardiometabolic derangements are observed in female rodents exposed to early life stress.

## 6 Early life stress primes the adrenal-adipose tissue axis in female rodents

Adipose tissue, composed of adipocytes, preadipocytes, and various resident immune cell populations, has come to light in recent years as a dynamic endocrine organ (100). Adrenal hormones, particularly aldosterone and corticosterone, have direct effects on adipose tissue homeostasis via the mineralocorticoid and glucocorticoid receptors, respectively. In turn, adipose tissue-secreted adipokines such as leptin and angiotensin II stimulate adrenal steroidogenesis (101–105) (**Figure 2**). Exacerbated adipose tissue expansion in obese MSEW female mice is associated with increased visceral adipose tissue-derived adipokines, showing that the adrenal-adipose tissue axis is sensitized in response to early life stress.

### 6.1 Aldosterone

Plasma aldosterone concentration and its robust association with obesity and metabolic syndromes has been well-reviewed elsewhere (106). This is particularly true in women, as visceral fat mass positively correlates with aldosterone and negatively correlates with insulin sensitivity in women, but not in men (107). More recently, a Chinese cohort demonstrated that increased neck circumference, an indicator of visceral body fat, is associated with elevated aldosterone in women (26). Another study showed that adults who reported childhood trauma had increased aldosterone, while trauma experienced for the first time in adulthood did not elicit a significant response (23). Childhood trauma exposure was associated with increased plasma aldosterone, particularly in individuals who reported physical or emotional maltreatment (22). While additional clinical and population-based studies are needed to strengthen the relationship between aldosterone and ACEs, findings from human studies correlate with rodent models of early life stress, highlighting their value in elucidating potentially translational therapeutics.

Female MSEW mice fed a HFD show exacerbated obesity and aldosterone production compared with diet-matched control mice, while chronic treatment in adulthood with spironolactone, a mineralocorticoid receptor antagonist, resulted in loss of fat mass coupled with increased glycerol efflux and decreased adipocyte size in MSEW females, but not in HFD controls (24). Tissue explants showed that in obese MSEW female mice, the source of increased aldosterone is predominantly the adrenal gland, although adipose tissue has a local renin-angiotensin-aldosterone system (RAAS) capable of synthesizing the hormone (24). Thus, adrenal-derived aldosterone may be mediating obesogenic effects through two candidate targets within adipose tissue, as illustrated in **Figure 2**. First, the MR expressed in adipocytes, the principal cell type of adipose tissue, could be driving transcriptional alterations in the cell leading to hypertrophy as a consequence of triglyceride accumulation. Second, the MR expressed in resident adipose-tissue dendritic cells can alter the pro-inflammatory milieu of the tissue promoting a similar phenotype.

#### 6.1.1 Adipocyte MR

Over-expression and over-activation of the adipocyte MR is directly related to obesity and metabolic syndrome-like phenotype in pre-clinical models. In an adipocyte-specific inducible MR overexpression model, Urbanet and colleagues found that adipocyte-MR upregulation correlated with progressive increases in body weight, visceral adipose tissue mass, and adipocyte size over 12 weeks without differences in food or calorie intake (108). Additionally, these mice showed markers of metabolic dysregulation, such as insulin resistance, hypertriglyceridemia, and hypercholesterolemia. Moreover, the metabolic dysregulation was further exacerbated by HFD (108). In primary mouse adipocyte cultures, MR stimulation with aldosterone resulted in an increased pro-inflammatory and obesogenic adipokine profile including elevated leptin, IL-6, PAI-1, and chemerin (109). Notably, MR-knockout adipocytes completely failed to accumulate lipids and had a 90% reduction in fatty acid binding protein aP2 (FABP4) expression, a carrier protein for fatty acids that promotes lipid storage. Similarly, Urbanet et al. found that aldosterone acts through lipocalin-like prostaglandin D2 synthase (PTGDS) downstream of the adipocyte MR to increase leptin, FABP4, and PPARy expression. While later studies showed that knocking out MR in adipocytes did not significantly change the metabolic phenotype of diet-induced obese male mice, further studies are needed to elucidate its role in the MSEW cardiometabolic phenotype, particularly in females. Notably, in female mice, adipocyte-derived leptin is a positive regulator of aldosterone synthesis in the *zona glomerulosa* of the adrenal glands (104) independent of obesity.

#### 6.1.2 Dendritic cell MR

Dendritic cells are a population of antigen-presenting immune cells that influence the phenotypic differentiation of T-cells (110). Aldosterone activates MR in dendritic cells and induces secretion of IL-6, and TGF-b, which promotes the polarization of naive T-cells to T-helper 17 (Th17) cells (111). Dendritic cells which are resident in adipose tissue are directly correlated to increased Th17 responses, obesity, and insulin resistance in both mice and clinical populations (112–114). In obesity, dendritic cells are abundant in visceral adipose tissue, and are particularly dense in fat-associated lymphoid clusters of murine gonadal adipose tissue (115). Blockade of dendritic cell migration into visceral adipose tissue prevents diet-induced weight gain and metabolic disturbances (115). Mice with loss of function specifically in fat-resident dendritic cells were similarly protected (116). In human adipose tissue, CD1c, a dendritic cell marker, has been positively associated with insulin resistance (113), and murine CD11c+CD64- dendritic cells are specifically cited as independent contributors to obesity and insulin resistance in diet-induced obesity models (112).

### 6.2 Leptin production during obesity

Leptin is an anorexigenic peptide hormone primarily produced by the adipose tissue (117) and is elevated in obesity (118). Leptin dampens ACTH-mediated steroidogenesis in the adrenal cortex (58), but is also an independent stimulator of aldosterone (104). Leptin deficient mice (ob/ob) have disrupted hypothalamic networks between the arcuate nucleus and the PVN that are only restored by leptin administration during the SHRP (119). MD in rat pups at PND10 abolishes the plasma leptin surge, while corticosterone, aldosterone, and ACTH are increased (58). Three- or twelve-hour MD in PND8 mouse pups was not sufficient to induce changes in leptin or corticosterone levels compared to non-handled mice (120, 121). Interestingly, leptin stimulation in primary adrenal cultures from PND10 MD rat pups produced more aldosterone than non-separated cultures (58).

Female rat offspring exposed to both western diet and LBN showed higher increases in plasma leptin than males after weaning (121). Female rats exposed to MS and high fat, high sucrose diet had increased leptin mRNA in periovarian adipose tissue, in addition to increased PPARy mRNA, serum insulin, and HOMA index (122). Exogenous leptin administration to adult MS rats does not suppress food intake or weight gain as it does in non-handled rats (123). Further, leptin was elevated plasma of young adults who reported either childhood maltreatment or parental loss compared to those who did not report early life stress exposure (124). Mechanistically, it has been shown that obese MSEW female mice showed increased gonadal white adipose tissue-derived leptin that was not observed in males or dietary controls. This increased leptin production was associated with Hypomethylated CpG sites in the leptin promoter region (29).

### 6.3 Angiotensin II

Angiotensin II (AngII) is a known for its actions as a vasoactive peptide regulating vascular tone and water and electrolyte homeostasis, as well as for being a pathogenic mediator of obesity-related comorbidities. Angiotensinogen is the sole precursor for AngII, abundantly produced by the liver, serving as substrate for renin to produce angiotensin I. Cleavage of Angiotensin I by angiotensin-converting enzyme 1 (ACE1). AngII is a potent stimulator of aldosterone from the adrenal glands (105), which mediates the sodium and water reabsorption in the distal bneprhon and renal collecting duct. As such, the expression of all the renin-angiotensin-aldosterone system (RAAS) componetns have been shown in several tissues, including brain, heart, vasculature and kidney. Yet, AngII can be generated in the adipose tissue by both local ACE1 cleavage, and through non-canonical generation by cathepsin D and cathepsin G (125, 126). AngII acts locally on adipocytes via the AT1 and AT2 receptors, directly regulating adipose tissue homeostasis (101). Anti-adipogenic effects have been observed, but also lipogenic effects including triglyceride accumulation and activation of fatty acid synthase and glycerol-3-phosphate dehydrogenase (127).

Female mice fed a HFD develop exacerbated adiposity that is associated with increased circulating AngII, most likely due to increased fat-derived AngII production in visceral adipose tissue (98). However, the pressor response to acute doses of AngII was similar between control and MSEW obese female mice before and after treatment with the ACE1 inhibitor enalapril, indicating that the sensitivity to this peptide is similar between MSEW and control mice. Conversely, male MSEW mice fed a HFD display similar adiposity and circulating AngII levels when compared to obese controls (98). Furthermore, blocking AngII production with enalapril lowered blood pressure in obese MSEW male mice without decreasing the sympathetic activation (98). Taken together, this data suggests that, in the settings of obesity, MSEW activates the RAAS in a sex-specific manner. Thus, AngII could play an important role in obesity expansion experienced by female mice exposed to early life stress.

## 7 Discussion

Epidemiological studies on ACE exposures that are not stratified by sex still typically find an association with BMI. In women, this association is often found to show a “dose-dependent” effect, where each additional reported ACE exposure contributes to further increases in BMI (13, 14). Male mice exposed to early life stress do not show exacerbated weight-gain as their female counterparts, likely because current behavioral paradigms do not reach a persistently high enough level of stress to recapitulate the cumulative effect of ACEs needed to promote increases in adiposity. Additionally, male offspring may require exposure to different types of stress during the SHRP to elicit a physiological response that mimics what is observed in humans.

The balance between adrenal steroid production and adipokine production is likely a key mediator of obesity in women who have experienced ACEs. Aldosterone from the adrenal gland drives the expression of leptin and other key adipogenic mediators in the adipose tissue, as well as promoting a broad pro-inflammatory milieu. Similarly, leptin is an important neuroendocrine mediator that feeds back on the hypothalamus, as well as the adrenal gland. Leptin plays an inhibitory role on the PVN of the hypothalamus, suppressing HPA axis activation (128). Studies have found that leptin increases aldosterone production from the adrenal gland (104), but also that it dampens the effects of ACTH on production of aldosterone and corticosterone (58, 129). It is likely that both increased leptin and increased aldosterone perpetuate one another in conditions of metabolic stress.

There are emerging areas of study that may shed light in the mechanisms underlying sex-specific shaping of the HPA sensitivity in response to early life stress. Steenblock et al. have characterized a distinct population of stress-responsive adrenocortical precursor cells marked by positivity for nestin (Nes+) (130). This population resides in quiescence under the adrenal capsule, and migrates centripetally in response to stress, differentiating into steroidogenic cells in their fated zones (130). Isolated *in vitro* cultures of these cells show a propensity to become aldosterone-producing when stimulated. Though sex differences in Nes+ cell activation have not been explored, aldosterone-producing cells may become enriched long-term in the adrenal glands of female rodents exposed to early life stress due to their innate stress susceptibility.

GLP-1 is a gut-derived peptide that activates the HPA axis, whereas infusion of GLP-1 in healthy adults reduced aldosterone in a small clinical cohort (131). GLP-1 receptor agonists such as Ozempic (semaglutide) and Trulicity (dulaglutide) have become massively popular drugs for the management of weight, diabetes, and cardiovascular health. To the authors’ best knowledge, there have been no trials evaluating the differential effects of GLP-1 receptor agonists in women exposed to ACEs. A small clinical study concluded that dulaglutide did not activate the HPA axis, however measurements were limited to cortisol measurements after dexamethasone suppression and ACTH stimulation. It is possible that an already-approved GLP-1 receptor agonist could target mechanisms linked to cardiometabolic dysregulation in women exposed to ACEs, opening new venus of precision medicine that may potentially aid this vulnerable population.

While various paradigms of early life stress in rodents and other animal models have been extensively studied, few research groups have emphasized obesity and metabolic disruptions as outcomes in any of these models. Considering the high prevalence of ACEs in the population and the robustness of these associations, further investigation of mechanistic approaches to cardiometabolic therapeutics could prove highly valuable to public health. This review highlights and discusses how the adipose tissue keeps the score of early life stress in the female body. Because prevention is often not a feasible strategy for mitigating the risks of early life stress exposures, continued animal research offers hope for targeted therapeutics that could help to even that score.
